# MiRNA-34c Regulates Bovine Sertoli Cell Proliferation, Gene Expression, and Apoptosis by Targeting the *AXL* Gene

**DOI:** 10.3390/ani11082393

**Published:** 2021-08-13

**Authors:** Hao Sun, Haibin Yu, Lixin Xia, Ping Jiang, Zitong Bai, Ming Gao, Zhihui Zhao, Runjun Yang, Xibi Fang

**Affiliations:** 1 College of Animal Science, Jilin University, Changchun 130062, China; sunhao12192021@163.com (H.S.); xialx18@mails.jlu.edu.cn (L.X.); baizitong96@163.com (Z.B.); gaoming930410@126.com (M.G.); 2 College of Coastal Agricultural Sciences, Guangdong Ocean University, Zhanjiang 524088, China; yuhb@gdou.edu.cn (H.Y.); jiangp@gdou.edu.cn (P.J.); zhzhao@gdou.edu.cn (Z.Z.)

**Keywords:** miR-34c, Sertoli cells, proliferation, synthesis, apoptosis, *AXL*

## Abstract

**Simple Summary:**

Fertility is one of the essential reproduction traits of bulls, and accurate prediction of fertility potential using a semen sample from a donor bull for artificial insemination is crucial to achieve consistently high reproductive efficiency. Somatic cells, such as Sertoli cells and Leydig cells, are important in testis formation and provide a nurturing and regulatory environment for spermatogenesis. Furthermore, it was suggested that non-coding RNAs, such as microRNAs, long non-coding RNAs, circular RNAs, and Piwi-interacting RNA, function as important regulators of gene expression at post-transcriptional level in spermatogenesis. In this study, microRNA-34c was verified to specifically regulate the *AXL* gene by targeting a sequence in the 3’ UTR; miRNA-34c can also influence the proliferation, apoptosis, and relative abundance of the transcript of male-reproduction-related genes. Therefore, microRNA-34c can be considered an essential regulator in the process of bull spermatogenesis. These results identify a key microRNA and functional genes in the process of cattle male reproduction, providing useful information for future marker-assisted selection of bulls with excellent sperm quality.

**Abstract:**

MicroRNAs (miRNAs) play significant roles in mammalian spermatogenesis. Sertoli cells can provide a stable microenvironment and nutritional factors for germ cells, thus playing a vital role in spermatogenesis. However, few studies elucidate the regulation of bovine testicular Sertoli cells by miRNAs. Here, we have reported that miRNA-34c (miR-34c) regulates proliferation, apoptosis, and relative transcripts abundance gene in bovine Sertoli cells. In bovine Sertoli cells, overexpression of miR-34c inhibited proliferation and relative abundance of gene transcripts while promoting apoptosis of Sertoli cells, and the effects were the opposite when miR-34c was knocked down. Receptor tyrosine kinase (*AXL*) was identified as a direct target gene of miR-34c in Sertoli cells, validated by analysis of the relative abundance of *AXL* transcript and dual-luciferase reporter assay. The relative abundance of the transcript of genes related to male reproduction in Sertoli cells was changed after the *AXL* gene was overexpressed, as demonstrated by the RT2 Profiler PCR Array results. In summary, miR-34c specifically regulated the *AXL* gene by targeting a sequence in the 3′-UTR, which could influence proliferation, apoptosis, and relative abundance of the transcript of male reproduction-related genes. Therefore, miR-34c could be considered an essential regulator in the process of bull spermatogenesis.

## 1. Introduction

MicroRNAs (miRNAs) belong to the small non-coding molecules that regulate the various biological process [1], including fat metabolism, biosynthesis, proliferation, apoptosis, and cell differentiation through targeting their genes [2,3,4,5]. miRNAs are present in spermatogonia, Sertoli cell and Leydig cells within the testis and are present in mature spermatozoa, which indicated miRNAs have important roles in the proper functioning of the male reproductive system [6,7,8].

MiR-34 consists of three members, including miR-34a, miR-34b, and miR-34c, highly evolutionarily conserved and belong to the same miRNA family [9,10]. According to our previous transcriptome sequencing data, miR-34c is one of the differentially expressed miRNAs between immature and mature bovine testes. The relative expression abundance in adult bovine testes is higher than immature testes in newborns. A previous study reported that miR-34c plays essential roles in many biological processes and can regulate proliferation and differentiation in many kinds of cells [11,12,13]. MiR-34c was reported to promote apoptosis by increasing the abundance of transcripts of various apoptosis-related genes in germline stem cells. This may impact the reproductive function of the goat [14]. Research results show that miR-34c can promote the differentiation of murine embryonic stem cells (mESCs) into male germ cells in mice [15]. Overall, many studies have demonstrated that miR-34c is involved in biological processes. However, there are relatively few studies on the effects of Sertoli cells in cattle, so in our research work, we explored the role of miR-34c in bovine Sertoli cells.

The testis is an organ where sperm cells are produced and contain various cells, including Sertoli cells, interstitial cells, and germ cells. Sertoli cells are irregular conical cells with prominent nucleoli and two satellite nucleosomes outside the nucleus [16]. Sertoli cells have various biological functions, such as basal tight junctions between adjacent Sertoli cells to form the blood-testis barrier, providing a stable microenvironment for germ cells, preventing toxins from entering the epithelium, and delivering energy to the sperm [17]. Sertoli cells can also secrete androgen-binding proteins (*ABPs*), and ABPs binds to androgens that are synthesized and secreted by interstitial cells, which, in turn, exert their biological functions [18]. Another vital role of Sertoli cells in the synthesis and secretion of cytokines, such as glial cell line-derived neurotrophic factor (*GDNF*) [19,20], bone morphogenetic protein 4 (*BMP4*) [21,22]. Inhibition of immature Sertoli cell proliferation may cause Sertoli cell-only syndrome (SCOS), germ cell dysplasia, and even azoospermia. Overall, immature Sertoli cells are closely related to germ cell development and spermatogenesis. Some studies have reported that the receptor tyrosine kinase (*AXL*) gene affects testicular immune function. It has been reported that *AXL* promotes zika virus (ZIKV) entry and negatively regulates the antiviral state of human Sertoli cells to augment ZIKV infection of the testes [23]. The previous study has shown that *AXL *in mouse Sertoli cells plays an important role in maintaining the stability of testicular immune function and the production of germ cells, which can affect mouse reproductive function [24]. However, there are relatively few studies on cattle reproduction. Therefore, this study investigated the effects of miR-34c on Sertoli cells, including cell proliferation, apoptosis, and secretory functions, and validated the target relationships with the *AXL* gene and the regulatory effect of the *AXL* gene on the relative transcript abundance of reproduction-related genes. This study provides the theoretical basis to improve cattle reproduction performance.

## 2. Materials and Methods

### 2.1. Ethics Statement 

According to the guidelines established by the Jilin University Animal Care and Use Committee the experiments were performed (Permit number: SY201901007). 

### 2.2. Cell Culture

The cells were isolated by the Animal Genetic Breeding and Reproduction Laboratory in the College of Animal Science of Jilin University. The cell identification results showed that immature bovine testicular Sertoli cells were successfully isolated. Briefly, we identified the isolated Sertoli cells by Feulgen staining, immunofluorescence, and detection of marker genes expression levels. The morphological structure of Sertoli cells was identified by Feulgen staining; Inhibin protein was detected by immunofluorescence staining. The superimposed image showed that inhibin protein was expressed in almost all Sertoli cells, which further showed that the isolated and purified Sertoli cells were close to 100%. Thus, the above experimental results can fully prove that the cells we isolated are Sertoli cells, and their purity can be close to 100%. The results of this study are published in our previous study [25]. After the vial of cells was removed from the liquid nitrogen storage, they were quickly placed in a 37 °C water bath for 3–5 min. When the cryotube liquid was thawed entirely, the cryotubes were centrifuged at 800 *g*/min for 8 min. Sertoli cells were incubated in DMEM/F12 medium (HyClone, Logan, OH, USA) containing 10% fetal bovine serum (FBS; HyClone, Logan, OH, USA) and 1% penicillin-streptomycin (HyClone, Logan, OH, USA) and maintained at 37 °C in a humidified 5% CO_2_ incubator (Thermo, Marietta, OH, USA). Then, Sertoli cells were cultured in a six-well plate. When the cell density reached approximately 75%, the vectors containing miR-34c mimics, miR-34c inhibitor, and negative control were transfected into the cells.

### 2.3. Vector Construction and Target Genes Prediction 

We obtained the coding sequence (CDS) of the *AXL* gene by nested polymerase chain reaction (nested PCR) (the primers for nested PCR are shown in Table 1) and then ligated the CDS region to the pBI-CMV3 vector (Clonetech, #631632, Mountain View, CA USA). The *AXL* gene overexpression vector was successfully constructed using the pBI-CMV3 expression vector. MiR-34c mimics, miR-34c inhibitors, and NC plasmids have been constructed by Gene Pharma (Suzhou, China). The miR-34c mimics capable of expressing the natural pre-miRNA sequence and the miR-34c inhibitor including the miRNA sponge sequence were used to up-regulate and down-regulate the expression level of miR-34c in cells, respectively. The specific sequence information was shown in Table S1. The DNA-binding sites and target genes of miR-34c were predicted by miRBase (Available online: http://www.mirbase.org (accessed on 19 May 2018)) [26] and TargetScan (Available online: http://www.targetscan.org (accessed on 19 May 2018)) [27].Potential binding site fragments of the 3′UTR of the *AXL* gene, whether the wild-type (WT) or a mutant (Mut) version, were inserted into the pmirGLO dual-luciferase reporter vector to construct the pmirGLO-*AXL*-WT and pmirGLO-*AXL*-mut vectors, which were verified to be correctly built. The insert sequences of the wild-type (WT) or a mutant (Mut) version were in Table S2.

### 2.4. Cell Transfection 

MiR-34c mimics, miR-34c inhibitor, Negative Control (NC), pBI-CMV3-*AXL*, or pBI-CMV3 were transfected into Sertoli cells using FuGENE HD Transfection Reagent (Prmega, Madison, WI, USA), with three replicates in each group. Each sample included 150 μL Opti-MEM (Gibico, Waltham, MA, USA), 7.5 μL FuGENE HD Transfection Reagent, and 3 μg vector, and incubates in a 1.5 mL centrifuge tube for 15 min at 25 °C. The pmirGLO-*AXL*-3′UTR-WT, pmirGLO-*AXL*-mut, and pmirGLO plasmids were co-transfected into Sertoli cells along with the miR-34c mimics, and each sample included 150 μL Opti-MEM, 7.5 μL FuGENE HD Transfection Reagent, and 3 μg vector, and incubate in a 1.5 mL centrifuge tube for 15 min at 25 °C. 

### 2.5. RNA Extraction and Real-Time Quantitative PCR (qRT-PCR)

The miR-34c mimics, inhibitor or NC, and the *AXL* overexpression vector were co-transfected into bovine Sertoli cells, and fluorescence was observed 24 h. RNAiso Plus (Takara, Dalian, China) was used to extract total RNA from the cells after 48 h, the spectrophotometer ND-2000 was used to measure the concentration and quality of total RNA, and 1 μg total RNA was reverse transcribed to cDNA by a PrimeScript™ RT Reagent Kit (Takara, Dalian, China), the first step (remove the genomic DNA): Add 2 μL 5 × gDNA Eraser Buffer, 1 μL gDNA Eraser and 1μg total RNA to 10 μL, 42 °C for 2 min; the second step: add 1 μL Prime Script RT Enzyme mix I and 1 μL RT Primer Mix/ specific miRNA primers, add 4 μL 5 × Prime Script Buffer 2 and 4 μL enzyme-free water, incubate at 37 °C for 15 min, 85 °C for 5 s. The qRT-PCR system was as follows: 5 μL FastStart Universal SYBR Green Master (Roche Diagnostics, Mannheim, Germany), 1 μL cDNA, 0.2 μL primer-F (10 μm/μL), 0.2 μL primer-R (10 μm/μL), and 3.6 μL RNase-free water. qRT-PCR was performed using PCRmax Eco 48 (PCRmax, Staffordshire, UK). Reactions were incubated at 95 °C for 10 min, followed by 40 cycles of 95 °C for 10 s and 60 °C for 30 s. Each sample was tested in triplicate. Quantitative primers for miR-34c, *AXL*, U6, *β-actin*, proliferating cell nuclear antigen (*PCNA*), *GDNF*, *BMP4*, C-X-C motif chemokine ligand 12 (*CXCL12*), BCL-2-Associated X (*BAX*), and PARP1 binding protein (*PARPBP*) were designed by Primer 6.0, and all the primers were synthesized by Sangon Biotech (Changchun, China). The details of primers for qRT-PCR are shown in Table 2. The Ct value of U6 small nuclear RNA was used as the internal control to assess the relative abundance of miR-34c. Additionally, *β-actin* gene was used as the internal control to assess the gene mRNA expression levels, respectively. MiR-34c and the relative abundance of the gene transcripts were calculated using the 2 − ΔΔCT method, and ΔΔCT = (Ct (positive) − Ct (reference)) − (Ct (control) − Ct (reference)) [28].

### 2.6. EdU Assay

For the EdU assay, Sertoli cells were cultured in 24-well plates, transfected with miR-34c mimics, inhibitor, or NC when the cell density reached approximately 70%. Fluorescence was observed after 24 h, and EdU was detected at 48 h. According to the manufacturer’s instructions of BeyoClick EdU-555 Cell Proliferation Assay Kit (Beyotime Biotechnology, Shanghai, China), a 20 μM EdU working solution was prepared. An equal volume was added to a 24-well plate, whereby the concentration of EdU in the 24-well plate was 10 μM incubation was continued for 2.5 h. The culture solution was removed, and the cells were fixed with 0.5 mL of 4% paraformaldehyde and then permeabilized with a permeabilizing solution. After using the Click reaction solution, the cells were incubated in the dark for 30 min. Nuclear staining was performed using Hoechst 33342 to facilitate observation of the proportion of cell proliferation detected. Observed under a microscope (Olympus, Tokyo, Japan) with a magnification of 100 times, it can be found that the cell nucleus is stained blue, while the proliferating cells are red.

### 2.7. Flow Cytometry

According to the manufacturer’s instructions, cell apoptosis rate was analyzed using flow cytometry (BD Biosciences, Heidelberg, Germany) with PE/7-AAD Apoptosis Detection Reagent Box (BD biosciences, San Diego, CA, USA). Sertoli cells were seeded in a six-well plate, cultured, and transfected with miR-34c mimics, inhibitor, or NC. Sertoli cells were harvested and washed with cold PBS twice. At room temperature, 100 μL of cell suspension was stained with 5 μL of PE/Annexin V and 7-aminoactinomycin (7-AAD) for about 25 min in the dark. The GCs were washed three times with cold 1 × phosphate-buffered saline. The laser wavelength of PE and 7AAD was set at 488 nm and 546 nm, respectively. The parameters of sheath rate were set at 80 μL/min. Each sample detected 10^5^ cells, and each sample was repeated three times. The cells were scored as follows: viable cells were PE/Annexin V- and 7-AAD-negative; cells that were in early apoptosis were PE/Annexin V-positive and 7-AAD-negative; cells that were in late apoptosis or already dead were both PE/Annexin V- and 7-AAD-positive. Data were analyzed using the BD FACSDiva™ Software v. 6.1.3 (BD Biosciences, Heidelberg, Germany).

### 2.8. TUNEL Assay

Sertoli cells were cultured in 24-well plates, transfected with miR-34c mimics, inhibitor, or NC when the cell density reached approximately 70%. Fluorescence was observed 24 h when the cells are 48 h after transfection on the culture plate. According to the manufacturer’s instructions, apoptotic Sertoli cells were detected using the One-step TUNEL apoptosis detection kit (Beyotime Biotechnology, Beijing, China). Nuclear staining was performed using Hoechst 33342 to facilitate the measurement of the proportion of apoptosis cells. We used a fluorescence microscope with a magnification of 100 times to observe about 12,000 cells in the field of view. The experiment consisted of 3 repeated experiments.

### 2.9. RT2 Profiler PCR Array Detection of the Relative Transcript Abundance of Reproduction-Related Genes

After 48 h of transfection with pBI-CMV3 (negative control) or pBI-CMV3-*AXL*, total RNA was extracted, and 3 μg of total RNA was reverse transcribed with RT^2^ First Strand Kit (Qiagen, Frederick, MD, USA). The reaction contained 3 μg total RNA, 2.0 μL 5 × gDNA Elimination Buffer, and DNase/RNase-Free Water up to 10 μL. After 42 °C for 5 min, a mix of 4 μL of 5 × RT Buffer3, 1 μL Primers and External Control Mix, 2 μL RT Enzyme Mix3, and 3 μL DNase/RNase-Free Water was added. The solution was incubated at 42 °C for 15 min, 95 °C for 5 min, and 91 μL DNase/RNase-Free Water was added. According to the manufacturer’s instructions, RT^2^ SYBR Green ROX qPCR Master Mix (Qiagen, Qiagen, Frederick, MD, USA) was used. Then, the RT2 Profiler PCR Array (CAPB13964, Qiagen, Frederick, MD, USA) was used to detect 80 genes related to reproduction. The RT2 Profiler PCR Array was carried out with the Mx3005p (Stratagene, La Jolla, CA, USA), and the reactions were incubated at 95 °C for 10 min followed by 40 cycles of 95 °C for 10 s and 60 °C for 1 min. Data of gene expressions were analyzed by RT2 Profiler PCR Array data analysis online software (Available online: https://geneglobe.qiagen.com/cn/analyze (accessed on 1 May 2021)).

### 2.10. Statistical Analysis

All experiments were carried out in triplicate, and statistical analysis was performed with GraphPad Prism 6 software and carried out by one-way analysis of variance (ANOVA) with Dunnett’s multiple comparisons tests and Student’s *t*-test. The results are expressed as the mean ± Standard Error of Mean (SEM), and the values were considered significant at * *p* < 0.05; ** *p* < 0.01; *** *p* < 0.001; and **** *p* < 0.0001.

## 3. Results

### 3.1. MiR-34c Inhibits Proliferation and Secretion in Sertoli Cells

**Figure 1:**
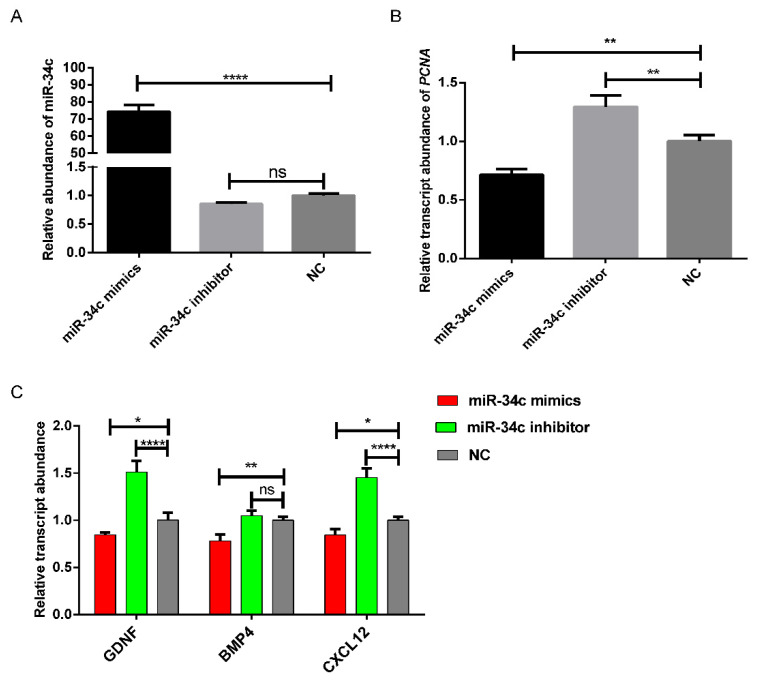
MiR-34c inhibits the proliferation-related gene expression in bovine Sertoli cells. (**A**) MiR-34c was upregulated in Sertoli cells after transfection with miR-34c mimics, and miR-34c was downregulated in Sertoli cells after transfection with the miR-34c inhibitor for 48 h. U6 was used as a reference. (**B**) Detection of the relative expression level of *PCNA* after transfection with miR-34c mimics, miR-34c inhibitor or Negative Control for 48 h. *β-actin* was used as a reference gene. (**C**) After transfection for 48 h, qRT-PCR was used to detect the relative abundance of the transcript of *GDNF*, *BMP4* and *CXCL12* in bovine Sertoli cells, and *β-actin* was used as a reference gene. * *p* < 0.05; ** *p* < 0.01; **** *p* <0.0001.

Experimental results showed that when miR-34c mimics were transfected, the relative abundance of the transcript of miR-34c was significantly increased (*p* < 0.0001). In contrast, the relative abundance of miR-34c was decreased when miR-34c inhibitors were transfected (Figure 1A). The qRT-PCR results for *PCNA* showed that miR-34c overexpression significantly decreased the relative abundance of *PCNA* mRNA (*p* < 0.01). In contrast, the relative transcript abundance of *PCNA* was significantly increased when miR-34c was inhibited (*p* < 0.01) (Figure 1B). The overexpression of miR-34c significantly decreased the relative transcript abundance of the Sertoli cell-secreted factors, including *GDNF*, *BMP4*, and *CXCL12* (*p* < 0.01) (Figure 1C). The inhibition of miR-34c significantly increased their relative transcript abundance of *GDNF* and *CXCL12* (*p* < 0.0001), and the relative abundance of *BMP4* mRNA not significantly changed (Figure 1C). 

**Figure 2:**
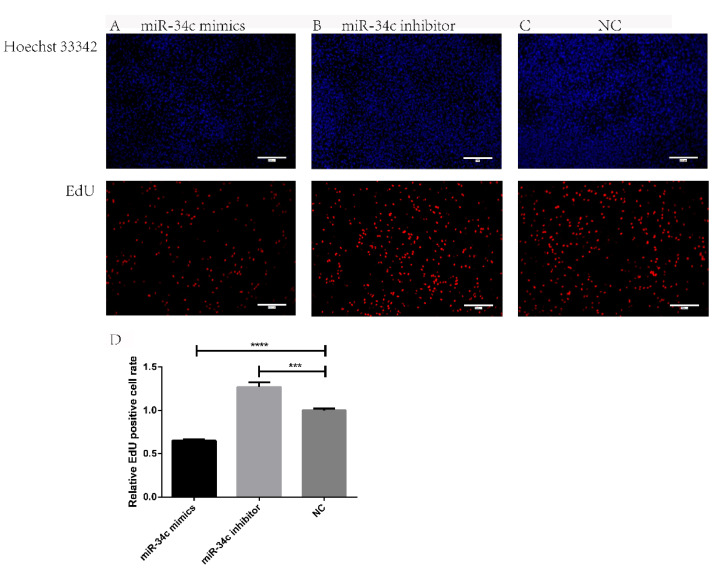
MiR-34c inhibits the proliferation of bovine Sertoli cells. (**A**–**C**) EdU and Hoechst 33342 staining of Sertoli cells. (**D**) Ralative EdU positive cell rate. The EdU-positive cells in bovine Sertoli cells after transfection and cell nuclei were counterstained with Hoechst 33342 (100×), Scale bar = 200 μm. Data were presented as means ± SEM. Standard Error of Mean (SEM) was indicated by error bars. *p* < 0.05 was considered to be statistically significant. *** p < 0.001; **** *p* < 0.0001.

DNA replication is inevitable in cell proliferation so that DNA synthesis can be used as a marker for Sertoli cell proliferation. EdU (5-Ethynyl-2′-deoxyuridine) is a thymidine analog, which can label newly formed DNA so that proliferating cells can be observed through a fluorescence microscope. The EdU staining results indicated that the number of positive cells was reduced in the miR-34c mimics group, indicating that the Sertoli cell proliferation rate was significantly decreased compared to the negative control group (*p* < 0.0001). The number of positive cells in the miR-34c inhibitor group was gained, indicating that the Sertoli cell proliferation rate was increased significantly compared to the negative control group (*p* < 0.001) (Figure 2). The above results indicate that miR-34c inhibits cell proliferation and the relative abundance of secreted transcription factors in bovine testicular Sertoli cells.

### 3.2. MiR-34c Increases the Apoptosis of Sertoli Cells

**Figure 3:**
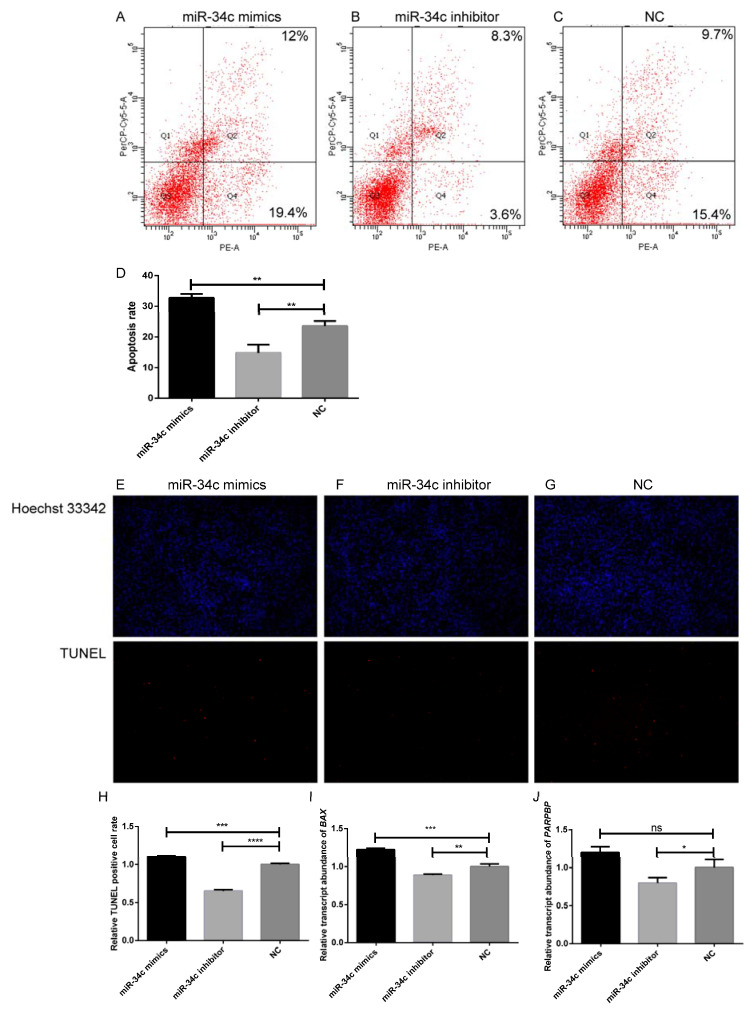
MiR-34c promotes the apoptosis of bovine Sertoli cells. (**A**–**D**) The rate of apoptosis in Sertoli cells was detected by flow cytometry, and apoptosis in Sertoli cells was analyzed by flow cytometry. (**E**–**H**) The TUNEL assay showed apoptotic cells among bovine Sertoli cells after transfection, and cell nuclei were counterstained with Hoechst 33342 (100×), Scale bar = 200 μm. (**I**,**J**) Data were presented as means ± SEM. Standard Error of Mean (SEM) was indicated by error bars. *p* < 0.05 was considered to be statistically significant. * p < 0.05; ** p < 0.01; *** p < 0.001; **** *p* < 0.0001.

The results after staining with PE/7-AAD showed that after miR-34c was upregulated, the apoptotic rate of bovine testicular Sertoli cells increased from 25.1% to 31.4%. In contrast, when miR-34c was inhibited, the apoptosis rate decreased from 25.1% to 11.9% (Figure 3A–D). As a cell undergoes apoptosis, specific phenomena can be observed, such as fragmented genomic DNA, which can be detected using the TUNEL assay. When miR-34c was upregulated, the apoptotic rate of bovine Sertoli cells increased, while when miR-34c was inhibited, the apoptotic rate was reduced (Figure 3E–H). Besides, overexpression of miR-34c could upregulate the relative transcript abundance of the apoptosis-related genes *BAX* and *PARPBP*. Simultaneously, the results were reversed when miR-34c was downregulated (Figure 3I,J), indicating that miR-34c could promote the relative transcript abundance of apoptotic genes in Sertoli cells.

### 3.3. Characterization of the Target Relationship of MiR-34c and AXL

**Figure 4:**
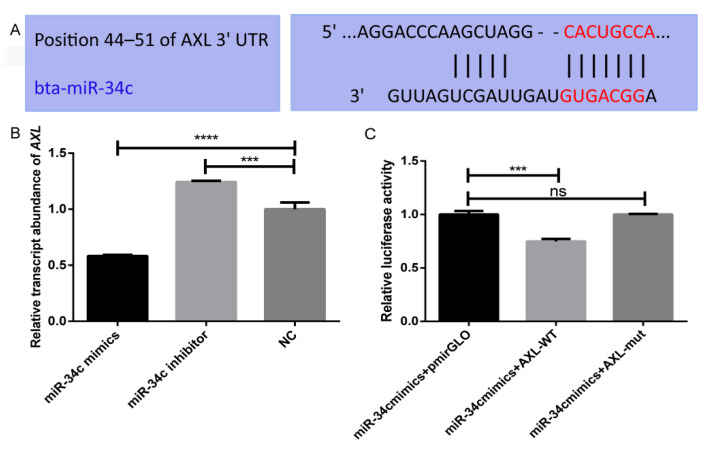
Verification of the target relationship between miR-34c and the *AXL* gene. (**A**) Sequence information of the binding site in the 3′UTR of *AXL* by TargetScanHuman 7.2. (**B**) The relative abundance of the transcript of the *AXL* gene. (**C**) Firefly and Renilla luciferase activities were measured after 48 h of transfection in Sertoli cells. *** *p* < 0.001; **** *p* < 0.0001.

The *AXL* gene is a direct target of miR-34c. Binding sequences for miR-34c were found in the 3′UTR of the *AXL* gene (Figure 4A). The qRT-PCR results showed that when miR-34c was overexpressed, the relative abundance of *AXL* mRNA was significantly decreased (*p* < 0.001), and the relative abundance of *AXL* mRNA was significantly increased (*p* < 0.01) when miR-34c was inhibited (Figure 4B). The result indicated that the relative transcript abundance of miR-34c and *AXL* were negatively correlated. The dual-luciferase reporter assay results showed that the miR-34c mimics and pmirGLO-*AXL*3′UTR-WT co-transfection group had significantly reduced luciferase activity (*p* < 0.001) compared to the miR-34c mimics and pmirGLO-*AXL*3′UTR-mut co-transfection group (Figure 4C). These results indicated that *AXL* is a direct target gene of miR-34c.

### 3.4. The Effect of the AXL Gene on the Relative Transcript Abundance of Marker Genes Related to Male Reproduction

**Figure 5:**
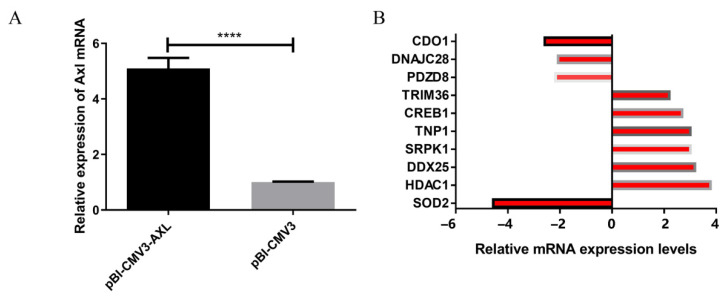
The effect of the *AXL* gene on male reproductive function genes. (**A**) The relative abundance of the transcript of *AXL* in Sertoli cells. (**B**) The relative abundance of the transcript of 10 male reproduction-related genes. Data were presented as means ± SEM. Standard Error of Mean (SEM) was indicated by error bars. *p* < 0.05 was considered to be statistically significant. **** *p* < 0.0001.

We constructed an *AXL* overexpression vector, and the relative abundance of the transcript of *AXL* increased significantly (*p* < 0.0001) post-transfection with pBI-CMV3-*AXL* (Figure 5A). The detecting genes of the RT2 Profiler PCR Array are essential to male animals’ reproductive function. The relative transcript abundance of 80 genes related to male reproduction was detected by the RT2 Profiler PCR Array. Among the 80 genes we tested, a total of 6 genes showed up-regulation, and four genes showed down-regulation (Fold change > 2 or <−2) (Table 3). The relative transcript abundance of tripartite motif containing 36 (*TRIM36*), histone deacetylase 1 (*HDAC1*), DEAD-box helicase 25 (*DDX25*), SRSF protein kinase 1 (*SRPK1*), transition protein 1 (*TNP1*)*,* and cAMP responsive element binding protein 1 (*CREB1*) was upregulated, while relative transcript abundance of cysteine dioxygenase type 1 (*CDO1*), superoxide dismutase 2 (*SOD2*), PDZ domain containing 8 (*PDZD8*)*,* and DnaJ member C28 (*DNAJC28*) was downregulated (Figure 5B). The analysis data of all 80 genes are shown in Table S3. The results showed that the *AXL* gene could affect the relative transcript abundance of reproductive marker genes. 

## 4. Discussion

Although many studies show that miR-34c plays a vital role in many life activities, few studies on whether miR-34c also has an essential function in bovine testes. In our present study, when miR-34c was overexpressed, Sertoli cell proliferation was inhibited, and Sertoli cell apoptosis was increased. Meanwhile, the proliferation was promoted, and apoptosis was decreased when miR-34c was inhibited. The number of normal sperm has a significant positive correlation with the number of mature Sertoli cells; each mature Sertoli cell, which is generated by immature Sertoli cell proliferation, can only support a limited number of germ cells for spermatogenesis, so the number of immature Sertoli cells also determines the reproductive capacity and spermatogenic efficiency of an individual animal [29,30,31]. Because a vital function of Sertoli cells provides a stable microenvironment, nutrition, and physical support for germ cells [32,33], and miR-34c can increase the apoptosis of bovine Sertoli cells and inhibit their proliferation, we speculate miR-34c may indirectly affect spermatogenesis by affecting the proliferation and apoptosis of Sertoli cells, but the specific results need further research. 

A critical function of supporting cells is to synthesize and secrete cytokines. In our results, when miR-34c was overexpressed, the relative transcript abundance of secreted factors *GDNF*, *BMP4,* and *CXCL12* was decreased; when miR-34c was knocked down, the relative transcript abundances of secreted factors *GDNF* and *CXCL12* was increased. It has been reported that the factor *GDNF* is critical for spermatogonial stem cell’s self-renewal and maintenance [19]. Studies have shown that *BMP4* can regulate diverse cellular responses, such as cell differentiation, migration, adhesion, and proliferation [34]. Besides, it has been reported that *CXCL12* can promote the growth of mouse spermatogonial stem cells [35]. Therefore, miR-34c may have an impact on spermatogonial stem cells of male animals by reducing the expression of these secreted factors directly or indirectly.

*AXL* is a member of the receptor tyrosine kinase subfamily, which includes three genes, TYRO3 protein tyrosine kinase (*TYRO3*), *AXL*, and MER proto-oncogene, and tyrosine kinase (*MERTK*) (TAM). Some studies indicated that *TYRO3*, *AXL*, and *MERTK* receptor tyrosine kinase triple knockout (TAM-/-) mice have male sterility due to impaired sperm development [15]. This result indicates that the *AXL* gene has a specific effect on the production of sperm. Studies have also shown that *AXL* plays a vital role in various cellular functions, including hematopoiesis, innate immune responses, platelet aggregation, phagocytosis of apoptotic cells, viral infection, vascular integrity, and permeability regulation, and cell survival [36]. In our results, after the *AXL* gene was overexpressed, the relative transcript abundances of *TRIM36*, *HDAC1*, *DDX25*, *SRPK1*, *TNP1,* and *CREB1* were increased. It has been reported that *TRIM36* deficiency causes morphological abnormalities in spermatozoa [37], and the *DDX25* gene plays a role in sperm maturation [38]. *TNP1* plays a crucial role in sperm nuclear condensation, and some studies have shown that *TNP1* is also essential for sperm formation [39,40]. *CREB1* is expressed during mitosis and spermatogenesis and is a necessary regulator of testicular development and spermatogenesis [41,42]. According to the results of previous studies, it is shown that male reproductive traits could benefit from *TRIM36* [37], *HDAC1* [43], *DDX25* [38], *TNP1* [39,40], *CREB1* [41,42], *CDO1* [44], *SOD2* [45] in mice or human, while *AXL* downregulated the relative transcript abundances of *CDO1* and *SOD2*. This result may be due to the genes highly expressed in spermatogonial stem cells or other cell types to improve male reproduction and differences in gene expression and regulatory mechanisms between different cell types. Therefore, we can speculate that the *AXL* gene can directly or indirectly affect spermatogenesis in male animals, but the specific results need further research.

## 5. Conclusions

**Figure 6:**
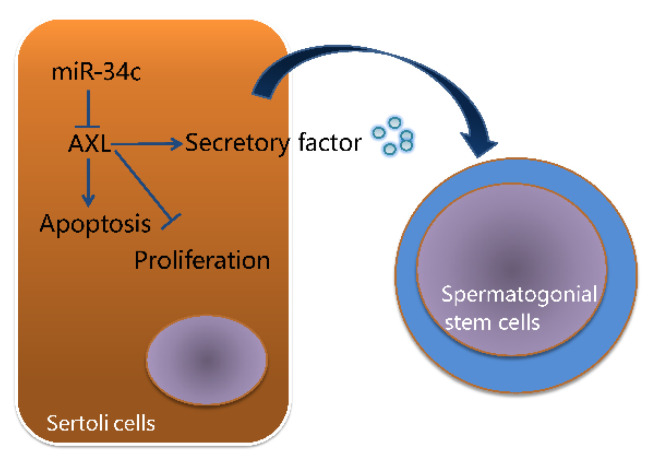
A schematic illustration of miR-34c targeting the *AXL* gene affects proliferation, apoptosis, and relative gene abundance of transcripts of reproduction-associated secreted factors in Sertoli cells.

This result demonstrates the effect of miR-34c targeting the *AXL* gene on proliferation, apoptosis, and relative gene abundance of transcripts of reproduction-associated secreted factors in primary bovine testicular Sertoli cells (Figure 6). Our study shows that miR-34c and *AXL* genes have a potential function on reproductive traits of bulls, and the result helps design excellent marker-assisted selection programs in bull breeding. 

## Figures and Tables

**Table 1:** The primers for Nested PCR.

Primer ID	Primer Sequence (5′–3′)	Product Length
*AXL*-1F	TACCAGACGAGCACGGAGA	3172 bp
*AXL*-1R	TCAGGCGCCGTCCTCCTG	
*AXL*-2F	ATGGGCAAGGTCCTGTTGGC	2664 bp
*AXL*-2R	TCAGGCGCCGTCCTCCTG	

**Table 2:** Primers of qRT-PCR.

Primer ID.	Primer Sequence (5′–3′)	Product Length
PCNA-F	CTCGTCTCATGTCTCCTTGGT	137 bp
PCNA-R	TGTCTTCATTGCCAGCACATT	
BAX-F	CGGAGATGAATTGGACAGTAACA	123 bp
BAX-R	CAGTTGAAGTTGCCGTCAGAA	
PARPBP-F	AGAGAATACGCAGTAGACGATGA	167 bp
PARPBP-R	AGAATATGAGCCAGAGCCAAGT	
GDNF-F	CTCACCGCCGTGCATCTAA	160 bp
GDNF-R	TGTCACTCACCAGCCTTCTAC	
BMP4-F	GGCTGGAATGACTGGATTGTG	105 bp
BMP4-R	GGCGTGGTTGGTTGAGTTG	
CXCL12-F	ATGCCCTTGCCGATTCTTTG	124 bp
CXCL12-R	CACTTGCCTATTGTTGTTCTTCAG	
*AXL*-F	CCGTTATGGAGAGGTGTT	107 bp
*AXL*-R	GTTCAAGGTGGCTTCAGT	
β-actin-F	AGAGCAAGAGAGGCATCC	103 bp
β-actin-R	TCGTTGTAGAAGGTGTGGT	

**Table 3:** Differently expressed genes related to male reproduction.

Gene Symbol	Fold Regulation	Expression
tripartite motif containing 36 (*TRIM36*)	2.2	up
histone deacetylase 1 (*HDAC1*)	3.78	up
DEAD-box helicase 25 (*DDX25*)	3.18	up
transition protein 1 (*TNP1*)	3.01	up
SRSF protein kinase 1 (*SRPK1*)	3.01	up
cAMP responsive element binding protein 1 (*CREB1*)	2.69	up
cysteine dioxygenase type 1 (*CDO1*)	−2.58	down
superoxide dismutase 2 (*SOD2*)	−4.56	down
PDZ domain containing 8 (*PDZD8*)	−2.16	down
DnaJ member C28 (*DNAJC28*)	−2.07	down

## Data Availability

Not applicable.

## Supplementary Materials

The following are available online at https://www.mdpi.com/article/10.3390/ani11082393/s1, Table S1: The insert sequences of miR-34c mimics, miR-34c inhibitor and NC, Table S2: The insert sequences of the wild-type (WT) and a mutant (Mut), Table S3: The expression fold changes of reproduction-related genes in bovine Sertoli cells.
