# Supplementary material for: MiRNA-34c Regulates Bovine Sertoli Cell Proliferation, Gene Expression, and Apoptosis by Targeting the AXL Gene
Source: Animals (Basel). 2021 Aug 13;11(8):2393. doi: 10.3390/ani11082393 (PMC8388803; doi:10.3390/ani11082393)
Supplement: Supplementary file 1 [file animals-11-02393-s001.zip › animals-1272266-supplementary.pdf]

**Table S1.** The insert sequences of miR-34c mimics, miR-34c inhibitor and NC.

| Sequence name | Vector information | Sequences |
|---|---|---|
| miR-34c mimics | pGCMV/EGFP/miR/Blasticidin | AATTCG**AGGCAGTGTAGTTAGCTGATTG**GTTT<br>TGGCCACTGACTGACCAATCAGCACTACACTGC<br>CTACCGG |
| miR-34c inhibitor | | AATTC**CAATCAGCTAACTACACTGCCT**CGAT**C**<br>**AATCAGCTAACTACACTGCCT**ACCGGT**CAATC**<br>**AGCTAACTACACTGCC**TTCAC**CAATCAGCTAA**<br>**CTACACTGCCT**TTTTTTACCGG |
| NC | | AATTCG**AAATGTACTGCGCGTGGAGAC**GTTTT<br>GGCCACTGACTGACGTCTCCACGCAGTACATTT<br>CACCGG |

Note: Bold represents the target sequence.

**Table S2.** The insert sequences of the wild-type (WT) and a mutant (Mut).

| Annealing sequence name | Sequences |
|---|---|
| AXL-WT-F | ctagtcctctcaggacccaagctaggcactgccactgggggacggccccctactt |
| AXL-WT-R | cgaagtagggggccgtcccccagtggcagtgcctagcttgggtcctgagagga |
| AXL-mut-F | ctagtcctctcaggacccaagctaggcaactacactgggggacggccccctactt |
| AXL-mut-R | cgaagtagggggccgtcccccagtgtagttgcctagcttgggtcctgagagga |

**Table S3.** The expression fold changes of reproduction-related genes in bovine Sertoli cells.

| Description | Symbol | Fold Regulation | Expression |
|---|---|---|---|
| Adhesion G protein-coupled receptor G2 | ADGRG2 | UNdetermined | |
| Aryl hydrocarbon receptor | AHR | UNdetermined | |
| A-kinase anchor protein 14-like | AKAP14 | UNdetermined | |
| A kinase (PRKA) anchor protein 4 | AKAP4 | -1.13 | down |
| Anti-Mullerian hormone | AMH | UNdetermined | |
| Apolipoprotein B | APOB | UNdetermined | |
| Bromodomain containing 2 | BRD2 | 1.04 | up |
| Bromodomain, testis-specific | BRDT | 1.27 | up |
| Calcium regulated heat stable protein 1, 24kDa | CARHSP1 | 1.40 | up |
| Cation channel, sperm associated 1 | CATSPER 1 | UNdetermined | |
| Cation channel sperm associated 4 | CATSPER 4 | UNdetermined | |
| Cyclin B1 | CCNB1 | UNdetermined | |
| CD46 molecule, complement regulatory protein | CD46 | -1.12 | down |
| Cysteine dioxygenase, type I | CDO1 | -2.58 | down |
| CAMP responsive element binding protein 1 | CREB1 | 2.69 | up |
| CAMP responsive element modulator | CREM | UNdetermined | |
| Cysteine-rich secretory protein 1 | CRISP1 | UNdetermined | |
| Cysteine-rich secretory protein 2 | CRISP2 | UNdetermined | |
| Cullin 3 | CUL3 | -1.13 | down |
| Cytochrome P450, family 19, subfamily A, polypeptide 1 | CYP19A1 | UNdetermined | |
| Deleted in azoospermia-like | DAZL | UNdetermined | |
| DEAD (Asp-Glu-Ala-Asp) box polypeptide 25 | DDX25 | 3.18 | up |
| DEAD (Asp-Glu-Ala-Asp) box polypeptide 4 | DDX4 | UNdetermined | |

| | | | |
|---|---|---|---|
| Doublesex and mab-3 related transcription factor 1 | DMRT1 | UNdetermined | |
| DnaJ (Hsp40) homolog, subfamily B, member 8 | DNAJB8 | UNdetermined | |
| Hypothetical LOC784171 | DNAJC28 | -2.07 | down |
| Eukaryotic translation initiation factor 2B, subunit 4 delta, 67kDa | EIF2B4 | 1.23 | up |
| Eukaryotic translation initiation factor 4 gamma, 3 | EIF4G3 | -1.26 | down |
| Epididymal sperm binding protein 1 | ELSPBP1 | UNdetermined | |
| Fas (TNF receptor superfamily, member 6) | FAS | 1.84 | up |
| F-box protein 5 | FBXO5 | UNdetermined | |
| FBJ murine osteosarcoma viral oncogene homolog | FOS | 1.79 | Up |
| GLI family zinc finger 3 | GLI3 | 1.44 | Up |
| Glutathione peroxidase 1 | GPX1 | 1.09 | up |
| Histone deacetylase 1 | HDAC1 | 3.78 | up |
| Heme oxygenase (decycling) 1 | HMOX1 | -1.01 | down |
| Hydroxysteroid (17-beta) dehydrogenase 7 | HSD17B7 | 1.31 | up |
| Heat shock transcription factor 2 | HSF2 | -1.82 | down |
| Heat shock 70kDa protein 4-like | HSPA4L | UNdetermined | |
| Interleukin 16 | IL16 | UNdetermined | |
| Kelch-like 10 (Drosophila) | KLHL10 | UNdetermined | |
| Laminin, alpha 5 | LAMA5 | 1.42 | up |
| Leptin | LEP | UNdetermined | |
| Luteinizing hormone/choriogonadotropin receptor | LHCGR | UNdetermined | |
| Lamin A/C | LMNA | 1.38 | up |
| Minichromosome maintenance complex component 8 | MCM8 | UNdetermined | |
| Myeloid/lymphoid or mixed-lineage leukemia (trithorax homolog, Drosophila); translocated to, 3 | MLLT3 | UNdetermined | |
| NADH dehydrogenase (ubiquinone) Fe-S protein 7, 20kDa (NADH-coenzyme Q reductase) | NDUFS7 | 1.74 | up |
| Niemann-Pick disease, type C2 | NPC2 | -1.37 | down |
| Outer dense fiber of sperm tails 1 | ODF1 | UNdetermined | |
| Parkinson protein 7 | PARK7 | 1.38 | up |
| Proprotein convertase subtilisin/kexin type 4 | PCSK4 | 1.71 | up |
| PDZ domain containing 8 | PDZD8 | -2.16 | down |
| Phosphoglycerate kinase 2 | PGK2 | UNdetermined | |
| Piwi-like 1 (Drosophila) | PIWIL1 | UNdetermined | |
| Phospholipase C, zeta 1 | PLCZ1 | UNdetermined | |
| Protamine 1 | PRM1 | UNdetermined | |
| Protamine 2 | PRM2 | UNdetermined | |
| Serpin peptidase inhibitor, clade A (alpha-1 antiproteinase, antitrypsin), member 5 | SERPINA 5 | UNdetermined | |
| Solute carrier family 25 (mitochondrial carrier; adenine nucleotide translocator), member 5 | SLC25A5 | -1.20 | down |
| Solute carrier family 26, member 8 | SLC26A8 | UNdetermined | |
| SWI/SNF related, matrix associated, actin dependent regulator of chromatin, subfamily a, member 2 | SMARCA 2 | 1.60 | up |
| Superoxide dismutase 1, soluble | SOD1 | 1.09 | up |
| Superoxide dismutase 2, mitochondrial | SOD2 | -4.56 | down |
| SPO11 meiotic protein covalently bound to DSB homolog (S. cerevisiae) | SPO11 | UNdetermined | |
| Spermatogenic leucine zipper 1 | SPZ1 | UNdetermined | |
| SRSF protein kinase 1 | SRPK1 | 3.01 | up |
| Stromal antigen 3 | STAG3 | UNdetermined | |

| | | | |
|---|---|---|---|
| Synaptonemal complex protein 3 | SYCP3 | 1.92 | up |
| TATA box binding protein | TBP | 1.26 | up |
| T-complex 11 | TCP11 | UNdetermined | |
| Testis expressed 11 | TEX11 | -1.17 | down |
| Transition protein 1 (during histone to protamine replacement) | TNP1 | 3.01 | up |
| Tumor protein D52-like 3 | TPD52L3 | UNdetermined | |
| Tripartite motif containing 36 | TRIM36 | 2.20 | up |
| Testis specific, 10 | TSGA10 | UNdetermined | |
| Ubiquitin associated protein 2 | UBAP2 | 1.69 | up |
| Ubiquitin carboxyl-terminal esterase L1 (ubiquitin thiolesterase) | UCHL1 | -1.18 | down |
| WAPL cohesin release factor | WAPL | 1.15 | up |
| Zona pellucida glycoprotein 3 (sperm receptor) | ZP3 | UNdetermined | |

UNdetermined: Gene expression level exceeds the minimum threshold and fold regulation cannot be calculated.
